# Publisher Correction: Moving event detection from LiDAR point streams

**DOI:** 10.1038/s41467-024-46400-x

**Published:** 2024-03-05

**Authors:** Huajie Wu, Yihang Li, Wei Xu, Fanze Kong, Fu Zhang

**Affiliations:** Department of Mechanical Engineering, The University of Hong Kong, Pokfulam, Hong Kong 999077 China

**Keywords:** Mechanical engineering, Electrical and electronic engineering

Correction to: *Nature Communications* 10.1038/s41467-023-44554-8, published online 06 January 2024

The Authors provide the corrected text for the following issues marked as points A and B,

A. **Error location:** Author list
  **Error:** Missing symbol of equal contribution specifying the equal author contributions for the first <Huajie Wu> and second author <Yihang Li>.
  **Correction text:** The original version of this Published Article omitted the symbol for the equal Authors contribution of the first two authors, <Huajie Wu> and <Yihang Li>. The correct way to display the Authors list is < Huajie Wu^1,2^, Yihang Li^1,2^, Wei Xu^1^, Fanze Kong^1^, Fu Zhang^1^> and a sentence should be added <^**2**^**These authors contributed equally**>.
B. **Error location:** Text

**Error:** Scientific error in multiple sentences, missing word **ref. in the points 1–4** (see below)

**Correction text:**

1. The original version of this Article contained an error in <location: Pg. 2, left column, the second last paragraph in Introduction>, which incorrectly read <In addition^19^, relies on a future frame for detecting moving objects, which exacerbates the delay further>. The correct version is <In addition, ref. 19 relies on a future frame for detecting moving objects, which exacerbates the delay further.>
2. The original version of this Article contained an error in <location: Pg. 2, left column, the second last paragraph in Introduction>, which incorrectly read ‘<Moreover^21,22,23^, are offline systems that require all LiDAR frames for occupancy map construction.>. The correct version is <Moreover, refs. 21,22,23 are offline systems that require all LiDAR frames for occupancy map construction.>
3. The original version of this Article contained an error in <location: Pg. 9, right column, the second paragraph in Discussion:>, which incorrectly read <In contrast, the performance of learning-based methods, such as^30^, could drop considerably if the test dataset is different from the training dataset.>. The correct version is <In contrast, the performance of learning-based methods, such as ref. 30, could drop considerably if the test dataset is different from the training dataset.>.
4. The original version of this Article contained an error in <location: Pg. 9, right column, the fourth paragraph in Discussion:>, which incorrectly read <Likewise, the occupancy map used in^20-23^ can address this ambiguity by distinguishing the unseen areas from seen ones.>. The correct version is <Likewise, the occupancy map used in refs. 20–23 can address this ambiguity by distinguishing the unseen areas from seen ones.>

