# Supplementary material for: Moving event detection from LiDAR point streams
Source: Nat Commun. 2024 Jan 6;15:345. doi: 10.1038/s41467-023-44554-8 (PMC10771495; doi:10.1038/s41467-023-44554-8)
Supplement: Supplementary file 1 — Supplementary Information [file 41467_2023_44554_MOESM1_ESM.pdf]

Moving Event Detection from LiDAR Point Streams

Huajie Wu[1†], Yihang Li[1†], Wei Xu[1], Fanze Kong[1] and Fu Zhang[1*]

[1]Department of Mechanical Engineering, The University of Hong Kong, Pokfulam, Hong Kong, 999077, China.

*Corresponding author(s). E-mail(s): fuzhang@hku.hk;
Contributing authors: wu2020@connect.hku.hk;
yhangli@connect.hku.hk; xuweii@connect.hku.hk;
kongfz@connect.hku.hk;
†These authors contributed equally to this work.

Supplementary Notes

1 More Discussions

1.1 M-detector as a LiDAR event detector

The point-by-point detection of M-detector along with the high sampling rate of a LiDAR sensor leads to a stream of event or non-event points at an interval of a few microseconds to sub-microseconds. This output of data is similar to event cameras, which generate a stream of events with a microsecond-level temporal resolution. We therefore can view the M-detector as a LiDAR event detector. Compared with event cameras that detect events at the hardware level (when sampling the pixel intensity), the LiDAR event detector is an algorithm operating on existing LiDAR measurements. Due to the compensation of ego-motion prior to the event detection, the output of M-detector naturally detects movements in an inertial frame. In contrast, the output of an event camera is in the camera body frame, which must be compensated in a subsequent software pipeline (e.g., [1]). Moreover, due to the active measurements of LiDAR sensors and the occlusion principle we exploited, a LiDAR event detector can detect moving objects in completely dark environments or with the same visual appearance as backgrounds. Finally, the very low false alarming rate (e.g., three out of a million points [2]) and accurate, direct depth measurements of a LiDAR sensor provide further values for autonomous robots.

As an event detector, the M-detector could be used in a variety of key robotic techniques. One is moving object detection and tracking, which are fundamentally important for robots' planning in dynamic environments. We demonstrated the use of M-detector in UAVs for avoidance of tossed balls. Due to the high computation efficiency of M-detector, the entire navigation pipeline, including event detection, localization, mapping, planning, and control, can run in real-time on the UAV onboard microcomputer. Moreover, M-detector had a low detection latency, which enabled the UAV to avoid the fast-approaching ball with a speed up to 7.6 m/s.

M-detector distinguishes points on moving objects from that on stationary objects, but it does not tell the object classes (e.g., cars, pedestrians). So an immediate subsequent step is feeding the labeled event points to a classifier (e.g., PointPillars [3], PointRCNN [4], and Part-$\mathbf{A}^2$ net [5]) for objects recognition. Experiment results (Supplementary Fig. 3) showed that the M-detector can recall 80% - 90% moving points while keeping only 10% - 15% of the raw points. The great point number reduction could significantly lower the computation time or resources required for object recognition. Although the classification of only event points gives up the recognition of stationary objects (e.g., parked cars), it often suffices the needs of planning tasks. In this task, stationary objects can be treated as part of the environment, so their avoidance can be achieved with conventional collision check (e.g., k-NN search [6] or occupancy inquiry [7]) without knowing the object class. In fact, it is not

possible to distinguish a movable stationary object from a non-movable object with the same shape (e.g., a statue or a model).

The event labels provided by M-detector could also increase the robustness of the subsequent objects classifier and the safety of the overall system. As a model-based method, M-detector is very robust to the objects' shape, size, and appearance without requiring massively labeled data. This ensures the M-detector to detect unusual moving objects (e.g., a tossed ball, see Fig. 5c and Supplementary Fig. 1) that did not appear in the training data hence failing the object recognition. For objects that had fewer samples in the training data (e.g., pedestrians and cyclists), M-detector could still detect them with high recall rates (e.g., 73% - 98% recall for pedestrians, 79% - 93% recall for cyclists, see Supplementary Fig. 3), which could be leveraged to improve the classifier performance on these objects.

A further application of M-detector is point completion, which aims to enhance the point density of a LiDAR frame by projecting points in previous frames. The density-enhanced frame could be re-sampled at different scanning patterns or viewpoints, enabling the data labeled for one LiDAR (e.g., multi-line spinning) or mounting position (e.g., rooftop) to be re-used for another (e.g., a non-repetitive scanning LiDAR carried by a drone). The data re-use is particularly important for emerging solid-state LiDARs [8, 9], whose scanning patterns could be different from existing ones preventing the use of existing labeled data or algorithms. One existing technique for point completion is scene flow estimation [10–18], which estimates a motion vector for each point in a LiDAR frame that indicates the displacement of the point from the last frame to the current one. Nevertheless, estimating the flow of all points in a frame is time-consuming and not necessary since most of the points are on stationary objects and can be projected to the next frame with the odometry information. A more sound approach is estimating the scene flow of only event points labeled by the M-detector, which could save much computation power due to the considerably fewer number of event points.

1.2 Limitations

One limitation of M-detector is that it only detects objects that are currently moving. Although this meets the goal of event detection, it could cause some undesired behaviors in practical applications. For example, in real environments, a moving object (e.g., car, pedestrian) could temporarily stop (e.g., at traffic signs), which will cause no event measurements at this moment (Fig. 5b). This issue can be overcome by tracking moving objects on raw points instead of event points only. In this way, objects that ever moved in the sensor FoV would be tracked constantly without a loss of track. For objects that never moved in the FoV, they could be well treated as part of the environment for collision check in a planning task.

Another major limitation of M-detector is the one-frame detection delay if the object moves along the LiDAR laser rays. In this case, the M-detector exploited the recursive occlusion principle to detect such movements (Fig. 3b,

c). However, the recursive occlusion principle requires at least two previous frames (e.g., at T_{k-1} and T_{k-2}), meaning that if the object began moving at T_{k-2}, the earliest frame it can be detected is the current frame instead of the previous one T_{k-1}. Fortunately, this delay does not cause major issues for robot planning due to two reasons: 1) it occurs only when the objects began moving, where the speed is quite low and the displacement between two frames is often very small. As a result, knowing them as moving or stationary plays little difference for robot planning which often has a certain safety clearance; 2) it occurs only for objects moving away from the LiDAR sensor, which further lowers the impact on the planning safety. For objects moving towards the LiDAR sensor, they meanwhile trigger the first occlusion principle (Fig. 3), which suffers from no such delay.

M-detector has an upper limit on the point number per LiDAR frame it can process in real-time. The upper limit is obtained by fitting the time consumption on existing datasets (see Fig. 6 and Supplementary Fig. 4) linearly, which is 167,845 points per frame at 10 Hz. This number is significantly higher than existing methods in comparison and also allows M-detector to be used with most of existing LiDARs, including Velodyne HDL-64E (130,000 points per frame), Waymo-64 (160,000 points per frame), Velodyne HDL-32E (80,000 points per frame), and Livox AVIA (24,000 points per frame), that have been widely used in applications including autonomous driving, UAV obstacle avoidance, traffic monitoring, surveillance and people counting, mapping, etc., as confirmed in the Results. Further improvement on this upper limit can be achieved by fully leveraging parallel computing provided by GPUs or CPUs. The more cores or threads in GPUs or CPUs can effectively lower the time consumption of M-detector by parallelizing the three tests and depth image construction.

M-detector cannot detect moving objects that are too close to static surroundings (Supplementary Fig. 5). The root reason is that in this case, it is not possible to distinguish if the exhibited occlusion behaviors are caused by the objects movements or by measurement noises. Nevertheless, the effect of this missing detection is very small for robot planning since with this small distance, the moving objects can be well treated as static environments in the planning module.

Although M-detector can detect the eventness of a point with microseconds latency, obtaining points on a moving object could cause a significant delay due to the LiDAR scanning. Existing LiDAR sensors are often scanning-type, where one or multiple laser pulses are driven to different directions to scan the FoV. Driving the laser pulses would take time, which causes a certain delay from the moment the object began moving in the FoV to the moment LiDAR samples measurements on it. M-detector cannot address this delay. Similarly, as an algorithm running on LiDAR measurements, M-detector suffers from the same disadvantages in size, weight, and power (SWaP) of LiDAR sensors.

Finally, there are a few limitations of M-detector that are caused by the interfaces of existing LiDARs. First, although M-detector could detect event

points right after they are sampled, existing LiDARs do not output an individual point upon sampling but accumulate it into a pack for output. This accumulation takes place within the LiDAR sensor hence cannot be removed by the M-detector, which is currently implemented on a standalone microcomputer. Second, the current implementation of M-detector cannot detect objects that cross the LiDAR laser rays without any background in presence (Fig. 3a). This is because existing LiDAR sensors often output the Cartesian coordinates of each point. In case of no background (or background beyond the measuring range) causing no returned laser pulse, the output point will be invalid for occlusion check. These two limitations could be overcome by a more tight integration of the M-detector with LiDAR sensors. For example, the M-detector could be implemented as a part of LiDAR sensor on its onboard processing chip, which enables the event detection to be performed in a true point-by-point manner. Running on the LiDAR onboard chip, the M-detector could also access raw information of a point, such as azimuthal angle, elevation angle, and range. In presence of no (or too far) background, the maximum detection range can be used for the occlusion check in the M-detector, which then detects moving objects as normal.

2 Detailed Explanation for M-detector's Workflow

As shown in Fig. 4a, except for the input and output modules, there are three main modules in M-detector, i.e., event detection, clustering & region growth, and depth image construction and maintenance.

For input, M-detector accepts either an individual point or a frame of points, with the sensor ego-motion compensated in advance. The result of ego-motion compensation can be provided by a LiDAR-based odometry or a full Simultaneous localization and mapping (SLAM) system. In the case of a frame input, the system serializes the frame into a stream of individual points, in which the points in a frame are arranged according to their sampling time. Then each point denoted in blue in Fig. 4a, whether input directly or serialized from a frame, is passed to event detection to determine whether it's an event point.

Event detection is performed to check if there are successful occlusions, which is the key component of M-detector. In this module, each point is required to undergo three parallel tests corresponding to the three occlusion principles (Fig. 3). The point is labeled as a moving event if any of the three tests is positive, denoted in red in Fig. 4a, which means there exist effective occlusions, or else labeled as a non-event point denoted in black in Fig. 4a. As shown in Fig. 4b, each point is projected into the latest N depth images stored in depth image library in turn to check if there is any effective occlusion. Test one is designed to detect whether the point is moving perpendicular to the laser ray (Fig. 3a). If there are more than M_1 depth images ($M_1 \leq N$) whose points are occluded by the current point $\mathbf{p}^{curr}$, the current point $\mathbf{p}^{curr}$

is regarded as moving perpendicular to the laser ray. Test two and three are designed to detect whether the point is moving parallel to the laser ray. Among them, test two is specifically for the point that is moving away from the LiDAR in parallel to the sensor laser ray (Fig. 3b), while test three is specifically for the point that is moving towards the LiDAR (Fig. 3c). If the current point $\mathbf{p}^{curr}$ is occluded by or occludes any points in all of the previous M_2 or M_3 depth images (denoted as $\mathbf{p}^{\mathbf{I}_{k-1}}, \cdots, \mathbf{p}^{\mathbf{I}_{k-M_2}}$ or $\mathbf{p}^{\mathbf{I}_{k-M_3}}$, respectively) and each of these points is occluded by or occludes all of its subsequent points (i.e., if $\mathbf{p}^{\mathbf{I}_{k-i}}$ is occluded by or occludes $\mathbf{p}^{\mathbf{I}_{k-j}}$ for all $i = 1, \cdots, M_2 - 1$ or $M_3 - 1$ and $j = i + 1, \cdots, M_2$ or M_3), the current point $\mathbf{p}^{curr}$ is regarded as moving parallel to the laser ray.

After each occlusion check, map consistency check, as introduced in the section of *Map consistency check*, is performed to check if the point is distant from stationary map points. Specifically, we perform map consistency check on multiple recent depth images along with each occlusion check. If there exist points in its neighborhood (i.e., $[-\varepsilon_\varphi, \varepsilon_\varphi]$ degrees in azimuthal angle, $[-\varepsilon_\theta, \varepsilon_\theta]$ degrees in polar angle, and $[-\varepsilon_f, \varepsilon_b]$ meters in depth), the current point is considered a stationary point and the occlusion check on this depth image (if positive) should be rejected. Otherwise, the occlusion check (if positive) on this depth map is regarded as an effective one.

Then, the currently labeled point denoted in red or black is accumulated for a certain time period, where clustering and region growth are then performed to improve the detection results by rejecting outliers and accepting inliers. In this step, accumulated points are voxelized (voxel size L_v) and then voxels containing event points are clustered. As introduced in the section of *Clustering and Region Growth*, isolated event voxels and their contained event points are rejected and all raw points in event voxels are labeled as events. This procedure can reject the static points falsely labeled as event points by event detection and bring back event points that were mislabeled as non-events.

The accumulated points with event labels revised by clustering & region growth are finally transformed into a depth image, which is saved in the depth image library for later use in event detection for future incoming points.

For output, if M-detector is in point-out mode, a point is output immediately after the event label is determined by the event detection module, leading to a detection delay equal to the processing time of the event detection module. If M-detector is in frame-out mode, the system output the event labels at each frame after clustering and region growth is completed. This mode of output increases the accuracy but introduces a longer delay due to the time required for point accumulation and clustering & region growth.

3 Success Rate

Success rate of moving object detection is important to many applications. To validate the object-level success rate of M-detector as well as to study its dominant affecting factors, we selected sequences from the *AVIA-Indoor* dataset that contains tossed balls of different sizes. We also collected additional

sequences with tossed balls, leading to a total number of 800 effective frames. All the frames were classified by ball size, distance to LiDAR, and distance to the nearest static surroundings. According to the ball size, we divided it into four categories, which are $\leq$0.10 m, 0.10-0.20 m, 0.20-0.25 m, and $\geq$0.25 m. Similarly, according to the distance to LiDAR, we divided it into three categories, which are 2-4 m, 4-6 m, and 6-8 m. The distance falling in 0-2 m was not considered because this LiDAR has a minimal measuring range of around 1 m and relatively large ranging errors within 2 m [2]. The maximum distance to LiDAR was 8 m limited by the range of the motion capture system. A moving ball in a frame was regarded as being successfully detected if at least 70% of its measurement points were detected by the M-detector (in frame-out mode).

Supplementary Fig. 5 shows the success rate of M-detector at different conditions. As seen from Supplementary Fig. 5a, the mean success rate was consistently high (ranging from 0.86 to 1.00) regardless of the ball size or its distance to the LiDAR sensor. In fact, the correlation between the ball size or location and success rate was rather weak. This phenomenon agreed well with the principle of the M-detector, where the occlusion between current points and background points holds stably regardless of the object size or location in the LiDAR FoV (Fig. 3). Instead, the occlusion could be considerably affected by the object's distance to its static surroundings. This property was verified by Supplementary Fig. 5b. When the ball was within 0.2 m of static surroundings, the success rate was relatively low (from 0.00 to 0.67). This is reasonable because the distance between the moving ball and surroundings was comparable to the LiDAR measurement noises and even smaller than the object size, where it is not possible to tell if the event was caused by true movements or by the measurement noises. When the distance was above 0.2 m, the mean success rate rapidly increased to 0.86 and then to 0.97 if the distance was further above 0.4 m. Meanwhile, the standard deviation decreased with this distance. When the moving ball was more than 0.7 m away from its nearest surroundings, the mean success rate increased to 1.00 with zero standard deviation (i.e., all moving balls were successfully detected), suggesting a consistently high success rate and robustness of the M-detector to object sizes and locations in this condition.

4 Different shape, color, and illumination

By exploiting the geometrical occlusions between current points and background points, M-detector is not just robust to object sizes and locations, but also to different shapes and colors. Moreover, since a LiDAR sensor is an active device, the measurements (hence detection performance) are robust to the illumination of the scene. These two properties were validated in the *AVIA-Indoor* dataset, which contained objects with different geometries, such as balls, bottles, boxes, frisbees, quadrotor, and pedestrians (Supplementary Fig. 1). These targets' sizes range from 5 cm to 180 cm. It also contained data collected in different illumination environments, with different number of objects or with

object colors similar to its background environments (Fig. 5c). In all these situations, the M-detector stably detected the moving objects (Supplementary Fig. 1 and Fig. 5c) in the scene as long as they are not very close to any static surroundings. Quantitatively, when the distance of the objects and static surroundings were greater than 0.2 m, the mean success rate reached 0.88 with a standard deviation of 0.10, among all different objects with various shapes, colors, and illumination conditions (Supplementary Fig. 6).

5 *AVIA-Indoor* Dataset

To assess the effectiveness of M-detector in detecting moving events from LiDARs with non-repetitive irregular scanning, we collected the *AVIA-Indoor* dataset. The dataset includes objects of varying shapes and sizes, as well as objects in different lighting conditions. Here, we describe how we set up the device, select the scenes and objects, and annotate the collected points to get the *AVIA-Indoor* dataset.

Sensors. A Livox AVIA LiDAR and a HIKROBOT camera are utilized to collect the data. The LiDAR boasts a Vertical Field of View (VFoV) of 77.2° and a Horizontal Field of View (HFoV) of 70.4°, while the camera's VFoV and HFoV are 66.5° and 82.9° respectively. The LiDAR recorded the first return of each laser pulse at a rate of 10 FPS (frame per second), while the camera captured images at 30 FPS. The two sensors were mounted in parallel on a table, with the camera used solely for reference purposes. The LiDAR frame is a right-hand coordinate system, with the z-axis pointing upward, the x-axis pointing forward, and the y-axis pointing left (Fig. 7b).

Scenes and objects. To obtain accurate object position data, data collection was conducted within a specific zone of an indoor laboratory. In order to test the robustness of M-detector under different lighting conditions, we also collected data in low-light environments and against a background that closely matched the color of the objects. To evaluate the M-detector's effectiveness in detecting objects of varying types, sizes, and shapes, we selected a diverse set of objects. These included four balls, two boxes, a frisbee, a bottle, a UAV, and two people, as depicted in Supplementary Fig. 1.

Labels. To obtain the labels for every point collected by the LiDAR, markers were affixed to each object. These markers were tracked by the motion capture system, which provided us with the precise position of the object's center. Using this center point as a reference, we searched for nearby points and labeled them as event points. This process enabled us to accurately identify and label each point in our dataset.

6 Labels for Open Datasets

In our experiments, three different LiDAR datasets, including *KITTI*, *Waymo*, and *nuScenes* (Supplementary Table 1), were utilized. However, these datasets did not provide event labels for every point, making it necessary for us to generate labels. We accomplished this by utilizing the annotations of the bounding

boxes provided in the datasets for the purpose of object detection and tracking: points in annotated objects whose speeds computed from two consecutive frames are higher than a given threshold (0.5 m/s for pedestrians and 1 m/s for vehicles and cyclists) are labeled as event points.

For *KITTI*, 10 out of the 11 training sequences offered for object tracking are selected in our study. The training dataset provided annotated bounding boxes for objects, along with tracking ID, location, and size information. Using the position difference between boxes with the same tracking ID in consecutive frames, the velocity of each box was estimated, and moving boxes with a velocity exceeding the manually prescribed threshold (adjusted according to different classes, e.g. 0.5 m/s for pedestrians, and 1.0 m/s for vehicles and cyclists) were selected. Points falling within the moving box were labeled as event points.

The *Waymo* dataset provided point cloud data from five different LiDARs. We only used the top LiDAR, which is Waymo's custom LiDAR and referred to as Waymo-64. We selected 45 out of the 798 training scenes available for object detection. The 3D bounding box labels with tracking IDs on LiDAR data were provided. To distinguish moving objects, we obtained the position of each box in different frames according to their tracking IDs and calculated their velocity based on the position difference between consecutive frames. Similar to the processing in *KITTI*, boxes with a velocity exceeding the manually prescribed threshold were considered moving objects. Points falling within those boxes were labeled as event points.

For *nuScenes*, we utilized the mini dataset consisting of 10 scenes. The attributes, including class ID, tracking ID, and velocity in three directions (x, y, z) for every point, were offered at 2 Hz. The velocity information of each point was utilized directly to classify whether a point is an event point. Similar to the processing in the two previous datasets, the points with velocity larger than the threshold are considered as event points. It's noted that the labels for event points were provided at 2 Hz while the raw data for event detection were collected at 20 Hz, so all evaluations in this dataset were performed only on frames where event labels were provided.

These three open datasets, together with the in-house dataset *AVIA-Indoor*, deploy different LiDARs and are of different features. Their information is summarized in Supplementary Table 1. Moreover, besides labeling the moving status of each point, we also labeled the tracking ID of each point by leveraging the tracking ID of the corresponding objects box provided by the dataset. This enabled us to classify different objects and evaluate the results at object level additionally, e.g., the metric of Recall-Compression (section 11).

7 Details of LMNet and SMOS

LMNet used RangeNet++ [19] or SalsaNext [20] as the backbone model for moving points segmentation. We used the SalsaNext backbone model due to its higher accuracy as reported in the original work [21] as well as found in

our actual experiments. The model was trained on *SemanticKITTI* dataset using sequences 00-10 with one residual image or eight residual images as its input (termed as LMNet-1 and LMNet-8*, respectively). For the latter case, the semantic information predicted by SalsaNext was also leveraged to correct the point segmentation while M-detector did not utilize this semantic information at all. The two variants, LMNet-1 and LMNet-8*, were also the two configurations used in the original work [21]. SMOS is an occupancy map-based method that classifies the moving or stationary points by voxels, so we label the points in their moving voxels as event points while the others as non-event points. To retain its best performance, we directly used the parameters they tuned on *KITTI* for the evaluation on dataset *KITTI*. For the other three datasets, SMOS failed to classify moving voxels using their tuned parameters due to different LiDAR sensors being used in these datasets. Therefore, we tuned their parameters on each dataset to our best efforts.

8 Instructions for Parameter Tuning

M-detector is a model-based method, so its parameters must be tuned for good performance. To facilitate its use in the future, some guidance on parameter tuning is provided in this section. Fourteen parameters are selected to illustrate how to choose the appropriate values, as shown in Supplementary Table 6, including the parameters for depth image construction (r_h and r_v), occlusion check (ε_h, ε_v, and ε_d), map consistency check (ε_φ, ε_θ, ε_f, and ε_b), clustering (L_v), and event tests (N, M_1, M_2, and M_3). The Precision-Recall curves are used for tuning and evaluation of each parameter. The computation for precision P and recall R is:

$$\mathrm{P} = \frac{\mathrm{TP}}{\mathrm{TP}+\mathrm{FP}}, \ \mathrm{R} = \frac{\mathrm{TP}}{\mathrm{TP}+\mathrm{FN}} \tag{1}$$

where TP, FP, FN denote the number of true positive, false positive, and false negative points on moving objects over all frames in the sequence used for tuning.

8.1 Guidelines for parameter tuning

Parameters for depth image construction. Parameters for depth image construction include the horizontal pixel size (i.e., resolution) r_h and the vertical pixel size (i.e., resolution) r_v. In M-detector, as introduced in the section of *Occlusion check on depth image*, the occlusion check is achieved based on depth images. To determine if there is occlusion, each pixel should contain at least one LiDAR point. Therefore, the resolution of the depth image r_h and r_v is usually slightly larger than (e.g., 1-2 times) the LiDAR horizontal and vertical angular resolution, respectively, ensuring that there are points in each pixel. If r_h or r_v is too small, pixels may have no points due to the limited LiDAR angular resolution, making it impossible to determine the occlusion nor eventness of a point projected into this pixel. The missing detection of event

points arising from this hence increases the FN. If the resolution is too large, a pixel will contain enough background points for occlusion determination, which helps the detection of event points and hence increases TP. Meanwhile, the large pixel size could also contain background points far from the test point, causing wrong event detection that increases FP.

Parameters for occlusion check. Parameters for occlusion check include the neighborhood size in the horizontal direction ε_h, the neighborhood size in the vertical direction ε_v, and the depth difference threshold ε_d. For ε_h or ε_v, since occlusion check is based on depth image, the value is usually set to 0.5 times r_h and r_v. For ε_d, the value is usually within 0.1-5.0 m. If the value ε_d is too small, event points can pass the occlusion check more easily, resulting in a higher number of TP. But meanwhile, static points could also be tested positive for the occlusion check due to the LiDAR ranging errors (often a few centimeters), which leads to the increment of FP as well. Conversely, if the value is too large that requires the current point to have a large depth difference from the background, points on moving objects with relatively low moving speed cannot be detected, which results in the increment of FN.

Parameters for map consistency check. Parameters for map consistency check include the neighborhood size in azimuthal angle ε_φ, the neighborhood size in polar angle ε_θ, the forward depth difference threshold ε_f, and the backward depth difference threshold ε_b. For ε_φ or ε_θ, the value is usually set to 2-8 times the horizontal or vertical angular resolution of LiDAR. In M-detector, as outlined in the section of *map consistency check*, map consistency check is based on the principle that points very close to static map points should also be considered static (i.e., the point should be rejected if it is tested as an event). These two parameters define the neighborhood size (defined on depth images) of the map points that a candidate point should be examined against. If these two parameters are very small, only the very nearby static map points can reject an event point, resulting in more TP points. Meanwhile, the small neighborhood is less likely to reject wrong event points, which results in more FP points as well. If ε_φ or ε_θ is large, more static map points from an extended neighborhood can reject an event point, making it less likely for event points to pass the map consistency check successfully, resulting in more FN points.

For ε_f or ε_b, the value is usually within 0.1-5.0 m. If the value is smaller, a smaller distance between the event point and static points will be allowed. Less static points are used to reject the positive occlusion. Thus, more event points will pass the map consistency check, resulting in a higher number of TP points. Meanwhile, the static points mislabeled as events are also easier to pass map consistency check, contributing to a higher number of FP points. Conversely, when the value is larger, the minimum distance between the current point and the static points should be kept at a larger value, which makes map consistency check reject more ineffective occlusions, resulting in a higher number of FN points.

Parameters for event tests. Parameters for event tests include the number of latest depth images saved in depth image library N, the minimum number of depth images required in test one M_1, the minimum number of depth images required in test two M_2, and the minimum number of depth images required in test three M_3. The value of N is often between 5-50, depending on the affordable computation resources as larger N requires more computation. For M_1, it must be greater than or equal to one while less than or equal to N. The suggested range for M_1 is 1-5. For M_2 or M_3, it must be greater than or equal to two to ensure consecutive occlusions while less than or equal to N. The suggested range for M_2 or M_3 is 2-5.

Generally speaking, with a larger number of depth images N, more information in the past is utilized during the event detection, so the possibility of a test point passing the occlusion tests will be higher, leading to an increase in TP and FP. For the effect of M_1, M_2, M_3, they are the minimum numbers of depth images required by the three tests, so a larger value will be more difficult for a test point to reach, resulting in a lower number of TP and FP points.

Parameter for clustering. The parameter for clustering includes the voxel size L_v in the voxelization process. This parameter is generally in the range of 0.1-1.0 m. As explained in the section of *Clustering and Region Growth*, clustering is performed on voxels containing event points (i.e., event voxels), then event points in isolated event voxels will be re-labeled as non-event points, and non-event points contained in voxel clusters of a certain number will also be re-labeled as event points. Reducing the voxel size will reduce the range for recalling non-event points, resulting in a decrease in TP. Conversely, with a larger voxel size, rejecting one isolated event pixel could reject more wrong event points (i.e., decrease in FP), but meanwhile reject more true event points contained in this voxel (i.e., increase in FN).

8.2 Verification and results

According to the analysis presented in the previous section, the parameters' values can be tuned and the results for datasets used in the benchmark are shown in Supplementary Table 2. Factually, we found that M-detector's performance is quite robust to the parameter values, requiring no precise tuning in practice. Taking the *AVIA-Indoor* dataset sequence 10 for example, we plot the Precision-Recall curves to investigate the performance change versus the parameter values. For each parameter under investigation, we perturb its values from its nominal values (i.e., the last row of Supplementary Table 2) while keeping all other parameters at their respective nominal values to obtain a Precision-Recall curve.

The Precision-Recall curves are presented in Supplementary Fig. 7 and Supplementary Fig. 8. It's noted that given that $\varepsilon_h/\varepsilon_v$ is usually set to 0.5 times the depth image resolution r_h/r_v, ε_b is usually set to ε_d, and the tuning methods of M_2 and M_3 are identical, so we only investigate the Precision-Recall for 10 independent parameters, including r_h, r_v, ε_d, ε_φ, ε_θ, ε_f, N, M_1, M_3, and L_v.

For parameters for depth image construction r_h and r_v, as illustrated in Supplementary Fig. 7, the 1x denoted the nominal value 0.30° and 0.30° respectively. The recall increased as the value became larger, indicating more TP points (note that the sum of TP points and FN points is fixed for a specific sequence), which agrees with the analysis in the previous section. Meanwhile, the precision slightly increased (in point-out mode) with the larger r_h and r_v too, which is caused by the larger increase in TP than in FP, a trend consistent with the previous analysis as well. Moreover, M-detector achieved relatively high performance (precision above 0.86 and recall above 0.7) in a large range of parameter values (0.5x-12.0x for r_h and 0.5x-6.0x for r_v).

For parameters for occlusion check ε_d, as illustrated in Supplementary Fig. 7, the 1x denoted the nominal value 0.3 m. With its value smaller, the recall increased, which means there are more TP points and fewer FN points, matched with the analysis in the previous section. The precision slightly increased because the increase rate of TP is slightly larger than FP's. Besides, M-detector maintained relatively high performance (precision above 0.86 and recall above 0.75) in a large range of parameter values (0.33x-16.67x for ε_d).

For parameters for map consistency check ε_φ, ε_θ, and ε_f, as illustrated in Supplementary Fig. 7, the 1x denoted the nominal value 0.6°, 1.2°, and 1.0 m, respectively. The recall increased and precision decreased with the value smaller, indicating more TP points, fewer FN points, and more FP points, which is consistent with the analysis in the previous section. Besides, M-detector maintained relatively high performance (precision above 0.75 and recall above 0.70) in a large range of parameters values (0.5x-10.0x for ε_φ, 0.25x-4.0x for ε_θ, and 0.1x-5.0x for ε_f).

For parameters for event tests N, M_1, and M_3, as illustrated in Supplementary Fig. 8, the 1x denoted the nominal value 5, 2, and 3, respectively. The recall increased and precision decreased as the value N was larger while M_1 and M_3 were smaller, indicating more TP points and fewer FN points, which verified the analysis in the previous section. Besides, M-detector maintained relatively high performance (precision above 0.82 and recall above 0.70) in a large range of parameter values (1x-10x for N, 0.5x-1.5x for M_1, and 0.67x-1.67x for M_3).

For the parameter for clustering L_v, as illustrated in Supplementary Fig. 8, the 1x base denoted the nominal value 0.30 m. As the value became larger, the recall initially increased and then decreased, which means TP increased first and then decreased while FN is verse, which agrees with the analysis in the previous section. For precision, it increased with the L_v at first, which means at those values, the clustering brought back more TP points than FP, corresponding with the analysis in the previous section for L_v. It was necessary to choose a suitable value for voxelization to ensure high recall while maintaining high precision. Also, M-detector maintained relatively high performance (precision above 0.88 and recall above 0.82) in a large range of the parameter value (0.17x and 3.33x for L_v).

In conclusion, the Precision-Recall curves in Supplementary Fig. 7 and Supplementary Fig. 8 demonstrated that M-detector can maintain relatively high performance over a relatively large range of parameter values, and the tuning curves for each parameter are consistent with the above analysis in Section 8.1.

8.3 The potential of adaptive thresholds

As the depth increases, objects at the boundary of the LiDAR could result in inconsistent depth measurements. One simple and straightforward solution to this problem is to truncate the points that are too far (with depth beyond a certain value). Such truncation does not harm the overall system performance in view of the long sensing range of a LiDAR sensor, where moving objects at far does not require timely detection and reaction of the ego-vehicle.

If it is truly desired to utilize the points at the boundary, a possible approach is to set different thresholds for different depth, considering that the depth measurements variation is caused by distance. To investigate the potential of adaptive thresholds, we implemented a method where different thresholds were assigned to ε_d, ε_f, and ε_b based on the distance of individual points. Usually, the greater the depth measurement variation is, the larger the depth comparison threshold should be to reduce the impact of such variation. Thus, in our implementation, we deployed a threshold that is linearly increasing with respect to the points distance, $\min(d_{max},\ d_i + \max(0,\ k_{thr} * (d - d_{thr})))$, where d_{max} is the maximum of adaptive threshold, d_i represents the initial depth threshold used in occlusion check (i.e., ε_d) or map consistency check (i.e., ε_f and ε_b), k_{thr} denotes the linear increasing rate, d_{thr} represents the distance the threshold starts increasing, and d is the distance of the tested point. When the point distance is below d_{thr}, a uniform threshold remains used. When the distance exceeds d_{thr}, the threshold increases linearly in proportion to the distance increment, with the increase rate determined by k_{thr}, until the maximum of threshold d_{max}.

To verify the effectiveness of this method, we performed tests on the *KITTI* dataset with the uniform parameters kept as the above nominal value. To determine suitable values for k_{thr}, d_{thr}, and d_{max}, several tests were conducted and the values of 0.1, 50, and, 6 were selected, respectively.

The detailed results comparing the performance of the adaptive threshold and uniform threshold in both point-out and frame-out modes are presented in Supplementary Table 7 and Supplementary Table 8. Overall, the adaptive threshold demonstrated better performance than the uniform threshold in point-out mode. Supplementary Table 7 clearly demonstrates a consistent improvement in the Intersection over Union (IoU). Out of the 20 sequences tested, the IoU increased in 17 of them and also the total IoU. However, in frame-out mode shown in Supplementary Table 8, the adaptive threshold either had no significant effect (in 8 out of 20 sequences) or even had a negative impact (in 10 out of 20 sequences), leading to an overall IoU comparable to that of the uniform threshold.

The above results can be explained as follows. Due to the linear increment from the initial threshold (the value taken by the uniform threshold strategy), the adaptive threshold has a larger threshold value, making it more difficult to be considered as positive (less FP and TP). In point-out mode, the eventness of each raw point is determined immediately after arrival while leveraging no extra information (e.g., point density), resulting in a certain number of FP points. Therefore, reducing such number of FP using adaptive thresholds could effectively bring up IoU (see Supplementary Fig. 9a and 9b). In contrast, in frame-out mode, a large number of FP points have already been eliminated by the step of clustering & region growth, so the benefits brought by adaptive threshold are not evident. Instead, the lower of TP caused by adaptive threshold could lower the overall IoU (see Supplementary Fig. 9c and 9d).

Based on the results and analysis provided, it is obvious that the adaptive threshold generally proves to be beneficial in point-out mode. However, in frame-out mode, the adaptive threshold either has no significant effect or even exhibits a negative impact on performance. Therefore, the specific choice of threshold depends on the specific requirements and objectives of the application.

9 Methodologies in the Application of UAV Obstacle Avoidance

The experiments on dynamic obstacle avoidance were validated using a small-scale quadrotor equipped with a Livox AVIA LiDAR for perception (50 Hz frame rate), a DJI Manifold2-C for computation with an Intel i7-8550 U CPU (1.8 GHz, 4 cores) and 8 GB RAM, and a CUAV Nora+ flight controller (Fig. 7a). The system consists of four main modules, namely state estimation, event detection, path planning (including dynamic obstacle avoidance during hovering and dynamic obstacle avoidance in forward flights), and trajectory tracking control. First, a frame of LiDAR points over 20 ms was sent to the state estimation module for ego-motion compensation and full state estimation. The ego-motion compensated frame is then passed to the event detection module to classify event points. Next, the points with event labels are used by the path planning module to plan an evasive trajectory according to the UAV's current state: at hovering or in forward flights. The planned trajectory is finally passed to the trajectory tracking controller to track. The system is implemented in C++ and runs in real-time on the DJI Manifold2-C. The trajectory tracking controller runs at 100 Hz, while the other three modules run at the frame rate of 50 Hz. The communication between the four modules is based on ROS Melodic running on Ubuntu 18.04, and the communication between the computer and flight controller is based on MAVROS [22].

State estimation. To estimate the odometry of the quadrotor and compensate the ego-motion of the input point cloud, we develop a state estimation module that tightly couples the input point cloud and inertial measurement unit (IMU) data in an on-manifold iterative Kalman filter framework [23]. Our

implementation is based on the FAST-LIO2 [24] framework, which is known for its fast, robust, and versatile LiDAR-inertial odometry estimation. However, we made modifications to reduce the latency in ego-motion compensation, which is required by the event detection module. Specifically, when a frame of LiDAR points is input to the module, the state predicted by IMU propagation at that time is used as the estimated odometry for point cloud ego-motion compensation. The ego-motion compensated point cloud is immediately sent to the event detection module without waiting for the Kalman update of the state or the map update in FAST-LIO2 [24]. This modification allows the state update and mapping to be processed in parallel with or after the event detection module (in our implementation, they are processed after event detection), thereby reducing the latency of ego-motion estimation and hence event detection. This ensures that the UAV can obtain the event detection result in time to plan its trajectory and avoid obstacles successfully.

Event detection. The ego-motion compensated point clouds are input into the M-detector to detect the event points. M-detector can find out all event points in low latency, e.g. 1.11 ms. This allows for efficient processing and real-time moving obstacle avoidance, which is crucial for safe and effective movement in highly dynamic environments.

Avoiding dynamic avoidance at hovering. When a UAV is hovering, it can avoid moving objects by simply selecting a new, safe target to move. During this process, event points are clustered using the DBSCAN algorithm [25] to identify the center of each cluster as the position of an obstacle. The incoming direction of the moving object can then be obtained as the direction connecting the position of the obstacle and the UAV's hover position. Then, a direction perpendicular to the incoming direction is selected as the new direction for UAV to move. Specifically, the new direction can be obtained by rotating the incoming direction around the body z-axis by 90 degrees. At the meantime, we select a prescribed distance (e.g. 1.5 m) as the moving distance along this direction. The avoidance strategy ensures the UAV can avoid moving obstacles. We generally make the UAV hover at a free space where no static obstacles existed within the prescribed distance for avoidance, and thus the UAV will not collide with static obstacles after moving the prescribed distance.

Avoiding dynamic avoidance in forward flights. This submodule consists of two parts: obstacle tracking and trajectory searching. The tracking part is designed to track the dynamic obstacle and predict its trajectory for collision check in the trajectory searching part. It has two steps: firstly, the DBSCAN algorithm [25] is utilized to cluster event points, and the centers of the obtained clusters are considered as the position of the observed dynamic object. Secondly, a Kalman filter is used to track and predict the trajectory of the dynamic object using a constant velocity model. In case of multiple moving objects detected, one Kalman filter is used to predict the future trajectory of one moving object. If event points measured on a dynamic obstacle are close to the predicted position at the current time, the measurement will be matched to the corresponding Kalman filter, and the positions of the obstacles in the

future (for a certain period, e.g., 2 s) are predicted and sent to the trajectory search part. If not, a new Kalman filter is initialized.

The trajectory searching part adopts a method introduced in the previous work [26] to find a trajectory for UAV, which builds two time-accumulated local k-d tree maps and utilizes the kinodynamic A* search algorithm to find a feasible trajectory to a target position. However, our method differs from the previous work in that we only use non-event points not all points to build the local map. Moreover, we utilize the predicted time, positions, and variances of trajectories of dynamic obstacles and the size of dynamic obstacles to build Gaussian Models, which are exploited to compute the probability of collision with dynamic obstacles. This collision probability is applied to check collision in the kinodynamic A* search algorithm to avoid moving obstacles. The resulting trajectory is passed to the trajectory tracking controller. Additionally, a replan mechanism will be triggered to search for a new safe and feasible trajectory if the UAV is far away from the currently tracked trajectory (i.e., 1 m) or if a moving obstacle may collide with the UAV (i.e., an object appears on the UAV's trajectory when the UAV passes there).

Trajectory tracking controller. This module comprises a cascaded PID controller, with the outer loop position controller deployed on the DJI Manifold2-C and the inner loop angular velocity controller deployed on the flight controller. The trajectory generated by the former module is first passed to the outer loop controller, where a PID controller [27] is used to compute the desired angular velocity and thrust. Then the resulting command is sent to the inner loop controller on the flight controller, enabling the UAV to avoid dynamic obstacles successfully.

10 Methodologies in the Application of Traffic Monitoring

One interesting application of event detection is traffic monitoring. To demonstrate the application, we used the sensor suite shown in Fig. 7b. The software pipeline processes each LiDAR frame as follows: event points labeled by event detection within a frame period (i.e., 100 ms) are clustered into instances. Each instance corresponds to a moving vehicle, such as a car, truck, bus, or motorcycle, and the instances are associated across consecutive frames to track their movement. To count the number of vehicles passing a particular area on the road, a counting line is virtually set on the road. Once a vehicle passes this line, the counted number is increased by one. The detailed implementation of this process is explained below:

Events clustering. To cluster event points, the first step is to voxelize them with a size of 0.3 m. Voxels containing event points are called event voxels and clustered using the DBSCAN algorithm [25]. Any isolated event voxels and their contained event points are removed. The resulting clustered event points in event voxels are grouped into several instances. To represent the region of each instance, the minimum axis-aligned bounding box (AABB) containing all

the event voxels in this cluster is extracted. The center position and size of the AABB correspond to the position and size of the instance, respectively.

Instance tracking. To associate instances across consecutive frames, Kalman filters are used for state prediction. The inter-frame displacement of each trajectory is predicted using a constant velocity model. To match current instances with the existing Kalman tracker, the Hungarian algorithm [28] is adopted. The algorithm calculates scores between current instances and predicted positions using the intersection volume over the union volume of the two bounding boxes. Once the current instances are matched to the predicted position with the maximum likelihood, the measurement of their position and size is used to update the corresponding Kalman filter for further association.

Vehicle counting. To determine if an instance has passed the counting line, we check if its position is on one side of the line while its previous position is on the other side. If this is the case, we can infer that the instance has passed the counting line, regardless of its moving direction. By tracking the number of instances that pass the line, we can obtain an estimate of the number of vehicles that have passed through a monitored location (such as a footbridge). This information can be useful for traffic management and planning.

11 Object-level Recall-Compression

We evaluate the object-level recall-compression of the proposed event detection method, M-detector, on *KITTI*, *nuScenes*, *Waymo*, and *AVIA-Indoor*. For each object class (e.g., vehicle, pedestrian, cyclist) in one of the above datasets, its recall is calculated by

$$\mathrm{R} = \frac{1}{n}\sum_{i=1}^{n}\frac{\mathrm{TP_i}}{\mathrm{TP_i}+\mathrm{FN_i}} \tag{2}$$

where n denotes the number of instances of this class. $\mathrm{TP_i}, \mathrm{FN_i}$ represent the number of true positive and false positive points of instance i, respectively. The compression is defined as the ratio of points labeled as non-events over the raw points

$$\mathrm{C} = \frac{\mathrm{TN}+\mathrm{FN}}{\mathrm{TP}+\mathrm{FP}+\mathrm{TN}+\mathrm{FN}} \tag{3}$$

where TN, FN, TP, and FP are true negative, false negative, true positive, and false positive points respectively without distinguishing instances. The compression measures the portion of the stationary points, which deserves less attention. A lower compression tends to label more raw points as events, which could bring higher computation time for subsequent processing. On the contrary, a higher compression labels less raw points as events, but may miss some true event points. A good detection should be able to obtain a high recall at high compression. We select several groups of parameters to obtain the Recall-Compression curve as shown in Supplementary Fig. 3.

Two results of the M-detector, both in point-out mode and frame-out mode, are presented. In *KITTI* dataset, recall of each class can reach 0.8 at around 0.9 compression. In *Waymo* and *nuScenes* datasets, recall of each class can

reach 0.8 at around 0.85 compression and 0.90 compression respectively. In *AVIA-Indoor* dataset, due to the structured indoor scene and the less ego-motion estimation error of the static LiDAR sensor, recall can reach almost 1.0 at around 0.94 compression. In principle, due to the impact of clustering and region growth, M-detector in frame-out mode finds missing event points back while rejecting outliers, which should achieve a higher compression or recall. But in actual implementation, clustering usually gives up an object if only several points on it are detected (e.g., the points on a car far away from LiDAR). This will cause the recall of this object to be zero, hence leading to the average recall of frame-out mode being lower than that of point-out mode, especially on the dataset *nuScenes*.

Supplementary Figures and Tables

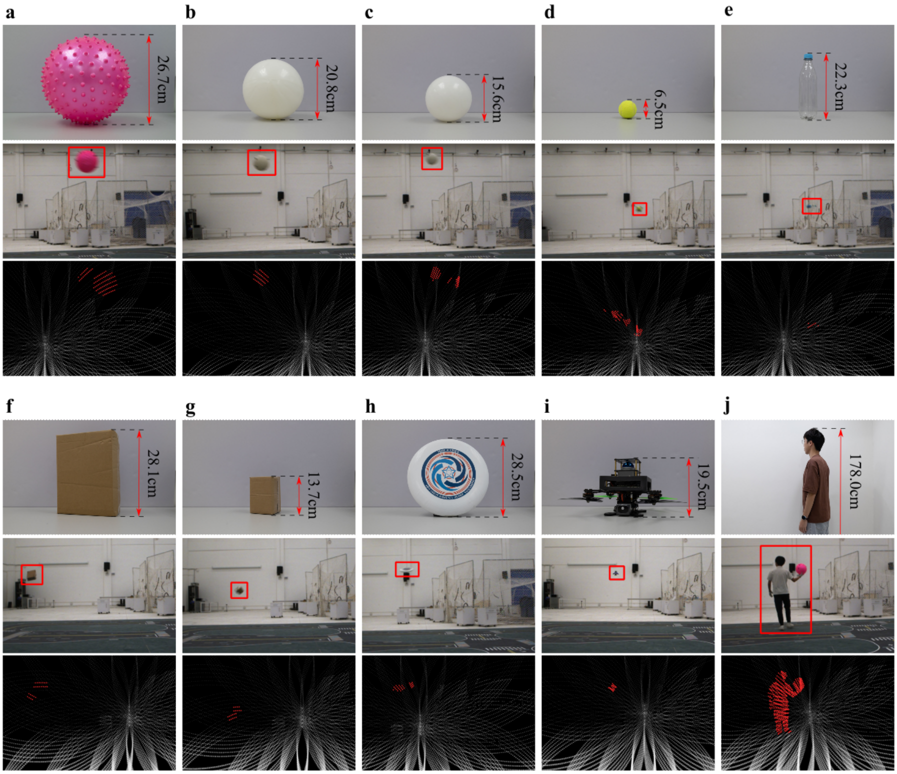

Supplementary Figure 1. The tossed objects in *AVIA-Indoor* dataset. There are ten groups. In each group, the first image shows the object's size, the second image shows the object after tossed (highlighted in the red box), and the third image shows the event detection result over one frame (red points represent event points and white points represent non-event points). **a** A pink ball with a diameter of 26.7 cm. **b** A white ball with a diameter of 20.8 cm. **c** A white ball with a diameter of 15.6 cm. **d** A tennis ball with a diameter of 6.5 cm. **e** A bottle with a height of 22.3 cm. **f** A box with a height of 28.1 cm. **g** A box with a height of 13.7 cm. **h** A frisbee with a diameter of 28.5 cm. **i** A UAV with a height of 19.5 cm. **j** A person with a height of 178.0 cm.

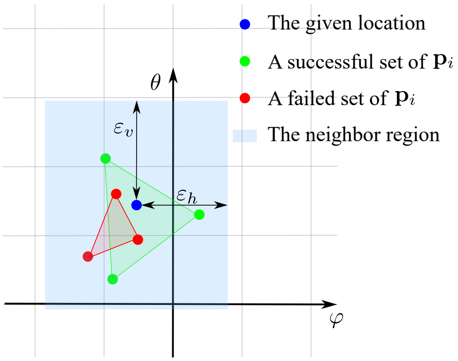

Supplementary Figure 2. **Illustration for depth interpolation in M-detector.** To interpolate the depth for the given location, the chosen points $\mathbf{p}_i$ $(i = 1, 2, 3)$ should fall into the neighbor region (the light blue area) and form a convex hull that contains the given location. The points in red are a failed set due to the violation of the latter condition, while the points in green form a successful set.

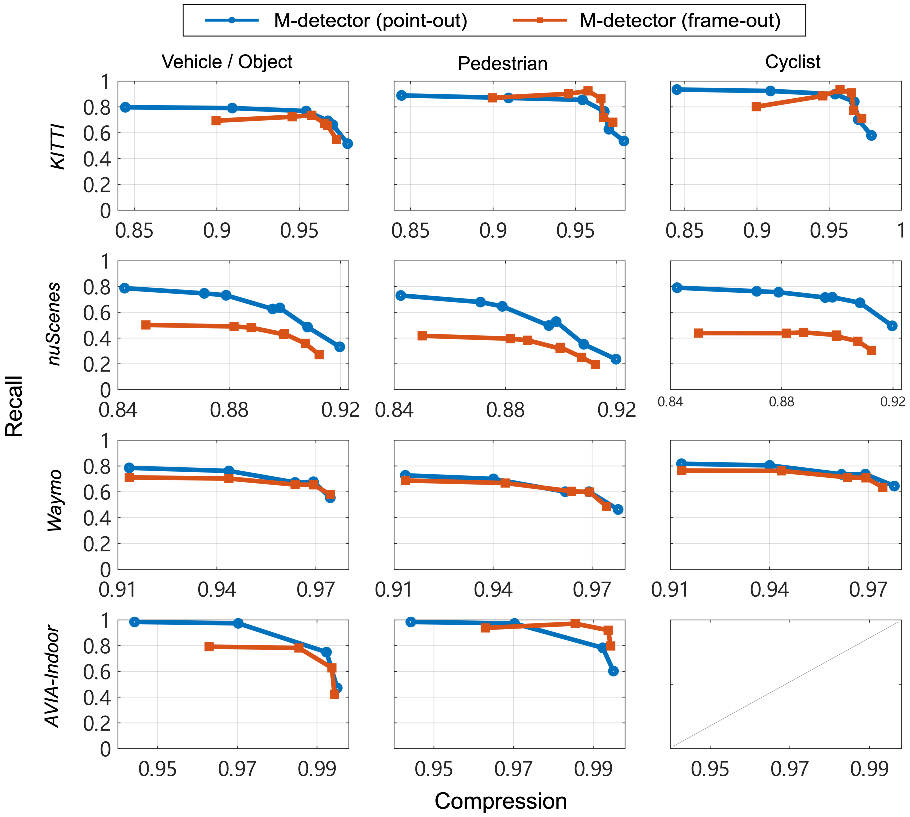

Supplementary Figure 3. **Recall-Compression curves of M-detector on different datasets**. Results in blue are obtained when the M-detector runs in point-out mode and results in red are obtained from the M-detector running in frame-out mode. Each data point represents the mean recall and compression rate averaged over all frames and sequences in that dataset. For datasets *KITTI*, *Waymo*, and *nuScenes*, they contain three classes: vehicles, pedestrians, and cyclists. For the dataset *AVIA-Indoor*, it contains tossed objects and pedestrians walking on the floor.

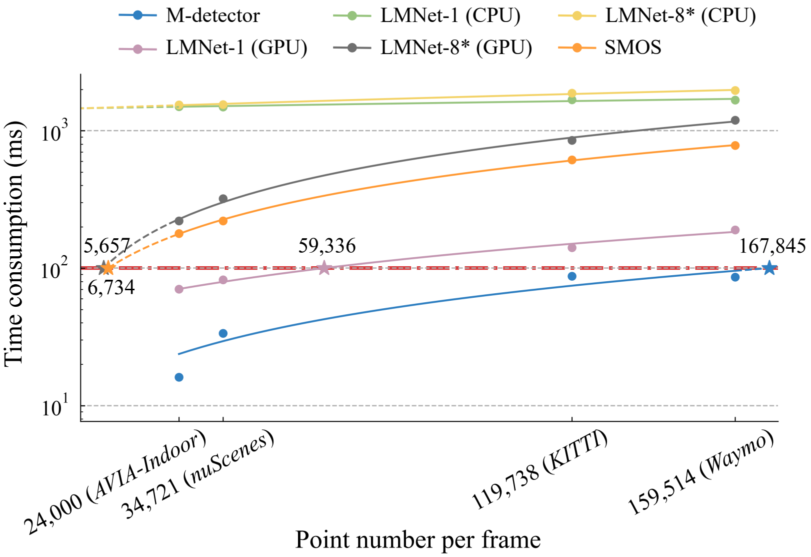

Supplementary Figure 4. **Per-frame time consumption of different methods versus the point number in a frame.** Each circle point in different colors denotes the time consumption of the corresponding method on that dataset with certain point numbers. These data are the same as the values tested in Section of *Time consumption and detection latency* in the manuscript. The upper limits (i.e., the star symbol) of different methods are obtained by linearly fitting the data of each method. Note that point number per frame is shown in the linear scale while time consumption per frame is shown in the log scale. The time is evaluated on a moderate computer with a central processing unit (CPU) of Intel i7-10700 (2.90 GHz, 8 cores) and 48 GB random access memory (RAM).

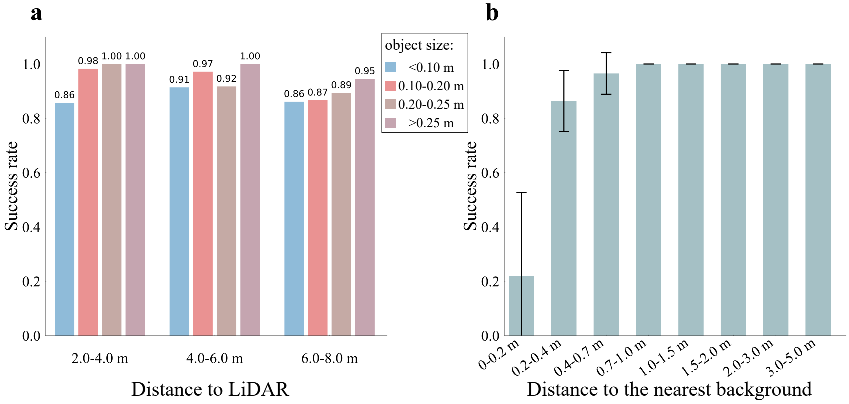

Supplementary Figure 5. **Results of success rate. a** The mean success rate with different ball sizes and the distances to LiDAR. **b** The mean and standard deviation of success rate with different distances to the nearest static background. Each bar denotes the mean success rate and the two edges around each bar represent one standard deviation among all balls (of different sizes and distances to LiDAR). Since the success rate is not a normal distribution, the value of mean plus one standard deviation could exceed one (e.g., the case of 0.4 - 0.7m in (**b**)).

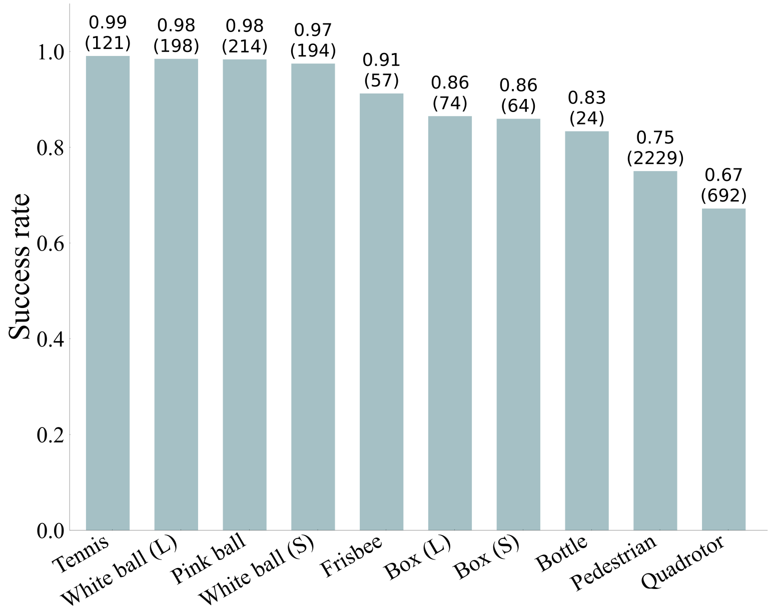

Supplementary Figure 6. **Success rate of M-detector on different objects.** Each bar denotes the success rate of the corresponding object. The numbers above the bar denote the success rate and the numbers in the parentheses denote the number of frames containing the corresponding moving object. The letter "L" in the parathesis of an object denotes a larger size of the object, and "S" denotes a smaller size. The quadrotor's success rate is relatively low because there are many frames where the quadrotor moved very slowly, which were all labeled as moving objects but triggered only partial events detection from M-detector (lower than 70%, the threshold for success detection). As for the number of frames, the quadrotor and pedestrian had effective frames way more than other tossed objects. This is because the quadrotor and pedestrian moved continuously (although could be very slow) while other tossed objects fell freely and appeared only in a few frames after the throw.

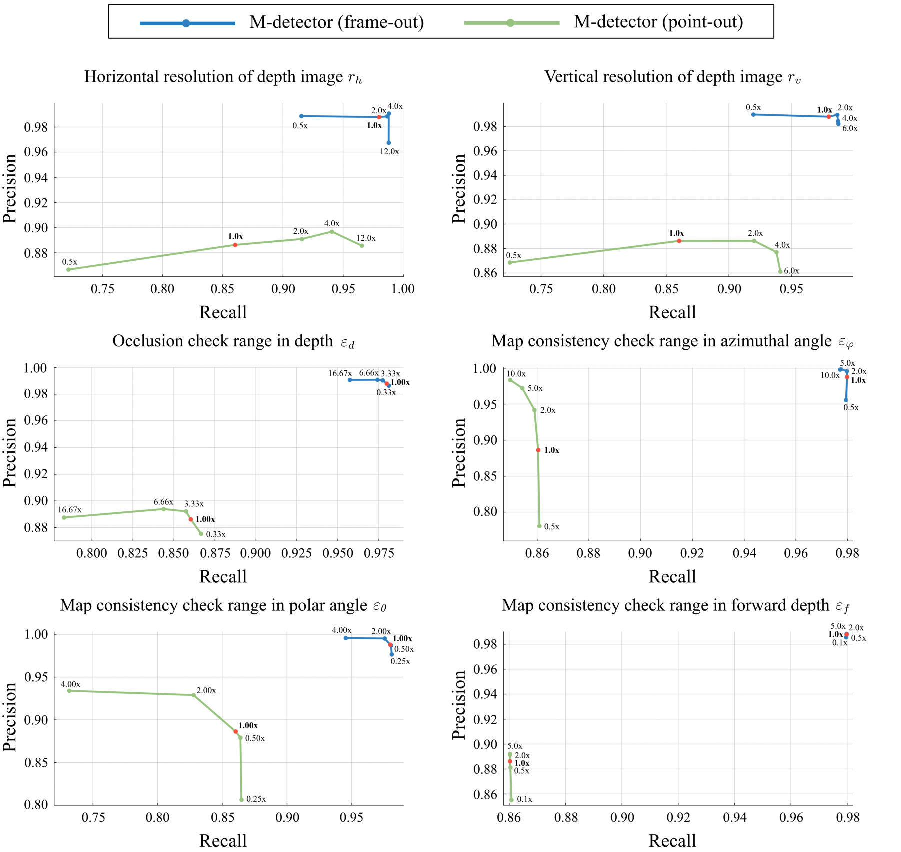

Supplementary Figure 7. **The first group of Precision-Recall curves of M-detector on six parameters.** Results in blue are obtained when the M-detector runs in frame-out mode and results in green are obtained from the M-detector running in point-out mode. Each data point represents the total precision and recall over all frames in sequence 10 of *AVIA-Indoor*. The label of each point represents the multiple of the parameter's current value relative to its nominal value (0.30° for r_h, 0.30° for r_v, 0.3 m for ε_d, 0.60° for ε_φ, 1.20° for ε_θ, and 1.0 m for ε_b).

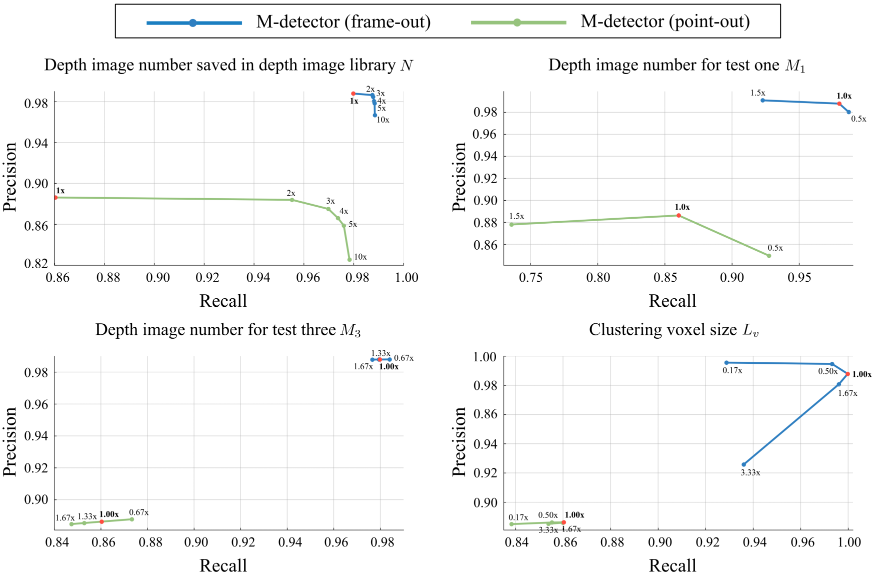

Supplementary Figure 8. The second group of Precision-Recall curves of M-detector on four parameters. Results in blue are obtained when the M-detector runs in frame-out mode and results in green are obtained from the M-detector running in point-out mode. Each data point represents the total precision and recall over all frames in sequence 10 of *AVIA-Indoor*. The label of each point represents the multiple of the parameter's current value relative to its nominal value (5 for N, 2 for M_1, 3 for M_3, and 0.3 m for L_v).

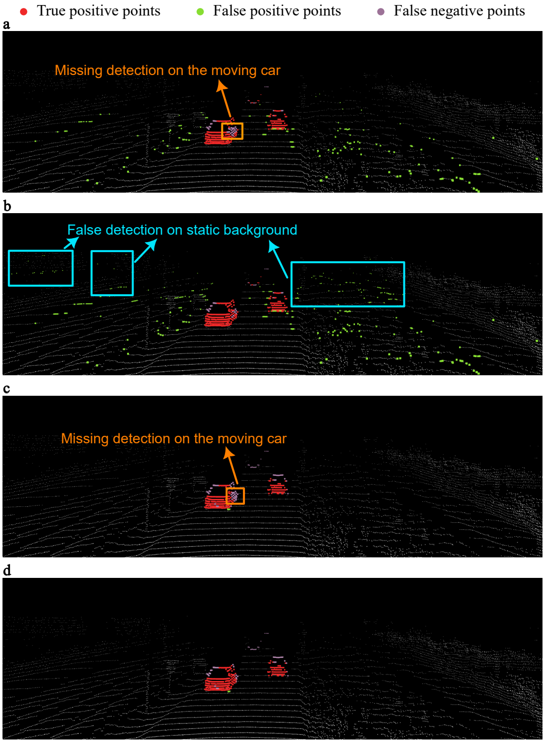

Supplementary Figure 9. **The comparison results between adaptive threshold and uniform threshold.** The point cloud is from the 207th frame in sequence 05 of *KITTI*. The red, green, and purple points denote true positive (TP), false positive (FP), and false negative (FN) points respectively. Boxes in detection results are manually labeled to highlight the regions of interest. **a** The detection result of adaptive threshold in point-out mode. **b** The detection result of uniform threshold in point-out mode. **c** The detection result of adaptive threshold in frame-out mode. **d** The detection result of uniform threshold in frame-out mode.

Supplementary Table 1. Summary of different datasets.

	LiDAR type	Frequency (Hz)	No. of sequences	Scene	No. of objects				No. of frames	Duration
					Vehicles	Pedestrians	Cyclists	Tossed objects		
KITTI	Velodyne HDL-64E	10	20	U,R,H[1]	13,676	10,306	1,459	0	7,561	12min34s
Waymo	Waymo-64[2]	10	44	U,R,H	50,440	84,323	1,587	0	8,730	14min59s
nuScenes	Velodyne HDL-32E	20	10	U,R	2,049	3,014	110	0	3,935	1min45s
AVIA-Indoor	Livox AVIA	10	45	L	0	4,480	0	4,654	13,491	22min29s
**Total**	-	-	119	U,R,H,L	66,165	102,123	3,156	4,654	33,717	51min47s

[1] U denotes urban, R denotes residential, H denotes highway, and L denotes indoor laboratory.

[2] Waymo dataset used a custom LiDAR whose resolution is equivalent to 64-line LiDAR but has a different vertical FoV and angular resolution.

Supplementary Table 2. Parameters of M-detector for different LiDARs contained in the datasets.

	Depth map		Occlusion check			Event detection				Map consistency				Clustering
	r_h	r_v	ε_h (n_h[1])	ε_v (n_v[1])	ε_d	N	M_1	M_2	M_3	ε_φ	ε_θ	ε_f	ε_b	L_v
Velodyne HDL-64E	0.29°	0.40°	0.29°	0.40°	0.5 m	15	2	2	3	0.57°	0.86°	0.5 m	0.15 m	0.3
Waymo-64	0.29°	0.57°	0.29°	0.57°	1.0 m	5	2	2	3	0.57°	0.57°	1.0 m	0.3 m	0.3
Velodyne HDL-32E	0.40°	1.26°	0.40°	1.26°	1.0 m	10	2	2	3	0.57°	1.72°	1.0 m	0.3 m	0.3
Livox AVIA	0.30°	0.30°	0.30°	0.30°	1.0 m	5	2	2	3	0.57°	1.15°	1.0 m	0.3 m	0.3

[1] $n_h = \lceil \varepsilon_h / r_h \rceil$, $n_v = \lceil \varepsilon_v / r_v \rceil$, where $\lceil . \rceil$ denotes the ceiling function.

Supplementary Table 3. Computation time breakdown of LMNet on different datasets.

	On CPU								On GPU							
	LMNet-1			LMNet-8*					LMNet-1			LMNet-8*				
	Residual Image[1] (ms)	Infer[2,3] (ms)	**Total** (ms)	Residual Image[1] (ms)	Infer[2,3] (ms)	Semantic (ms)	merge (ms)	**Total**[4] (ms)	Residual Image[1] (ms)	Infer[2,3] (ms)	**Total** (ms)	Residual Image[1] (ms)	Infer[2,3] (ms)	Semantic (ms)	merge (ms)	**Total**[4] (ms)
KITTI	43.2	1640.8	1684.0	294.2	1578.0	1507.6	4.6	1878.0	89.9	51.1	141.0	749.8	100.5	46.9	2.5	852.8
Waymo	56.4	1621.6	1678.0	387.1	1570.1	1447.1	5.6	1963.3	137.6	52.3	189.9	1067.9	119.9	47.5	4.3	1192.1
nuScenes	11.9	1478.9	1490.7	78.7	1456.9	1454.0	1.3	1549.1	34.3	48.2	82.5	262.0	55.9	44.8	2.0	320.0
AVIA-Indoor	14.0	1487.3	1501.4	63.8	1463.2	1473.7	1.7	1542.8	22.4	48.1	70.5	166.6	53.3	45.2	1.2	221.3

[1] The time consumption of residual image is a bit different from the value presented in [21]. After inquiring the author, we learned that the presented computation time in [21] was obtained based on implementation that was different from their open-sourced code, which was used to obtain the time consumption here.

[2] A mistake on counting the time consumption in SalsaNext [20] (the inference network of LMNet) was found. The number of frames they counted was twice of the actual numbers, which led to the time consumption they counted being around half of the actual value. The time consumption here was obtained after correcting this mistake.

[3] LMNet-8* used a different SalsaNext model from LMNet-1 for inference, hence having a different inference time.

[4] In LMNet-8*, the semantic segmentation ran in parallel to the event points inference, hence only the larger time consumption between semantic segmentation and inference contributed to the total time.

Supplementary Table 4. Computation time breakdown of event detection and clustering & region growth in M-detector on different datasets. In the table, the number represents the percentage of the time consumption of this part over the total detection latency (consisting of both event detection and clustering & region growth).

	Event detection			Clustering & region growth	
	Projection	Occlusion check	Map consistency check	Clustering	Region growth
KITTI	39.38%	20.17%	38.21%	1.99%	0.24%
Waymo	50.79%	10.03%	36.91%	1.92%	0.35%
nuScenes	68.07%	9.53%	19.01%	3.01%	0.38%
AVIA-Indoor	29.69%	15.64%	52.70%	1.92%	0.05%

Supplementary Table 5. The mean and standard deviation of the time consumption in different applications. In the table, the number before the symbol "±" represents the mean time and that after represents the standard deviation.

UAV obstacle avoidance			Traffic monitoring (ms)	Surveillance and people counting (ms)	Mapping (ms)
Event detection (ms)	Planning (ms)	Control (ms)			
1.11 ± 0.08	0.13 ± 0.06	0.03 ± 0.06	66.99 ± 43.97	47.73 ± 17.33	84.48 ± 8.89

Supplementary Table 6. Suggested ranges for parameters in M-detector.

Module	Symbol	Parameter explanation	Suggested range
Depth image construction	r_h	The horizontal pixel size (i.e., resolution) of the depth image	a_h-$2a_h$[1]
	r_v	The vertical pixel size (i.e., resolution) of the depth image	a_v-$2a_v$[2]
Occlusion check	ε_h	The neighborhood size in the horizontal direction	$0.5a_h$-a_h
	ε_v	The neighborhood size in the vertical direction	$0.5a_v$-a_v
	ε_d	The depth difference threshold	0.1-5.0 m
Map consistency check	ε_φ	The neighborhood size in azimuthal angle	$2a_h$-$8a_h$
	ε_θ	The neighborhood size in polar angle	$2a_v$-$8a_v$
	ε_f	The forward depth difference threshold	0.1-5.0 m
	ε_b	The backward depth difference threshold	0.1-5.0 m
Event tests	N	The number of latest depth images saved in depth image library	5-50
	M_1	The minimum number of depth images required in test one	1-5
	M_2	The minimum number of depth images required in test two	2-5
	M_3	The minimum number of depth images required in test three	2-5
Clustering	L_v	The voxel size	0.1-1.0 m

[1] a_h is the horizontal angular resolution of LiDAR.
[2] a_v is the vertical angular resolution of LiDAR.

Supplementary Table 7. Comparison of adaptive threshold and uniform threshold on *KITTI* dataset in point-out mode.

Sequences	Adaptive threshold				Uniform threshold			
	TP	FP	FN	IoU	TP	FP	FN	IoU
00	99,821	17,201	26,498	0.696	99,899	19,732	26,420	0.684
01	136,282	26,051	59,973	0.613	137,187	35,813	59,068	0.591
02	72,229	27,022	5,257	0.691	72,482	36,487	5,004	0.636
03	147,442	73,814	47,890	0.548	148,260	100688	47072	0.501
04	199,646	48,226	48,536	0.674	200,421	80,698	47,761	0.609
05	309,884	22,002	74,161	0.763	313,212	33,799	70,833	0.750
06	139,312	66,684	59,043	0.526	139,698	90,662	58,657	0.483
07	140,823	60,388	55,587	0.548	152,029	114,496	44,381	0.489
08	171,939	122,063	118,753	0.417	187,793	183,773	102,899	0.396
09	166,088	40,136	301,878	0.327	166,414	65,242	301,552	0.312
10	239,200	62,835	39,242	0.701	240,211	84,772	38,231	0.661
11	10,099	666	3,424	0.712	10465	1,109	3,058	0.715
12	121,929	37,475	29,691	0.645	122,083	51,410	29,537	0.601
13	19,450	13,280	3,499	0.537	19,924	16,863	3,025	0.500
14	150,268	21,203	14,878	0.806	151,209	29,902	13,937	0.775
15	320,406	12,772	46,148	0.845	320,419	19,149	46,135	0.831
16	248,111	28,491	22,184	0.830	248,111	28,738	22,184	0.830
17	461,347	61,131	115,235	0.723	462,426	81,298	114,156	0.703
18	1,375,009	162,899	473,763	0.684	1,375,836	180,200	472,936	0.678
19	997,415	126,472	1,313,366	0.409	1,022,951	162,074	1,287,830	0.414
Total	5,526,700	1,030,811	2,859,006	0.587	5,591,030	1,416,905	2,794,676	0.570

Supplementary Table 8. Comparison of adaptive threshold and uniform threshold on *KITTI* dataset in frame-out mode.

Sequences	Adaptive threshold				Uniform threshold			
	TP	FP	FN	IoU	TP	FP	FN	IoU
00	106,383	2,461	19,936	0.826	106,407	2,552	19,912	0.826
01	147,147	6,934	49,108	0.724	147,703	7,241	48,552	0.726
02	72,687	8,439	4,799	0.846	72,799	8,521	4,687	0.846
03	153,875	8,345	41,457	0.755	154,145	8,457	41,187	0.756
04	233,339	4,955	14,843	0.922	233,836	5,001	14,346	0.924
05	358,581	5,080	25,464	0.922	361,241	5648	22,804	0.927
06	168,067	13,032	30,288	0.795	168,204	13,427	30,151	0.794
07	139,504	2,728	56,906	0.700	149,958	4,401	46,452	0.747
08	188,688	9,706	102,004	0.628	198,740	12,072	91,952	0.656
09	188,146	5,049	279,820	0.400	187,225	5,659	280,741	0.400
10	257,819	14,488	20,623	0.880	258,236	14,952	20,206	0.880
11	10,121	635	3,402	0.715	10,222	647	3,301	0.721
12	129,192	14,736	22,428	0.777	129,301	15,730	22,319	0.773
13	1,8516	1,551	4,433	0.756	18,718	1,720	4,231	0.759
14	147,231	11,395	17,915	0.834	147,770	11,795	17,376	0.835
15	329,054	10,254	37,500	0.873	329,195	10,665	37,359	0.873
16	257,796	21,690	12,499	0.883	257,796	21,690	12,499	0.883
17	551,655	26,947	24,927	0.914	551,884	27,401	24,698	0.914
18	1,468,764	143,906	380,008	0.737	1,469,605	144,684	379,167	0.737
19	1,384,019	50,614	926,762	0.586	1,403,820	55,454	906,961	0.593
Total	6,310,584	362,945	2,075,122	0.721	6,356,805	377,717	2,028,901	0.725

Supplementary References

[1] Falanga, D., Kleber, K., Scaramuzza, D.: Dynamic obstacle avoidance for quadrotors with event cameras. Science Robotics **5**(40), 9712 (2020)

[2] LIVOX AVIA: USER MANNUAL. https://terra-1-g.djicdn.com/65c028cd298f4669a7f0e40e50ba1131/Download/Avia/Livox%20Avia%20User%20Manual%20202204.pdf

[3] Lang, A.H., Vora, S., Caesar, H., Zhou, L., Yang, J., Beijbom, O.: Pointpillars: Fast encoders for object detection from point clouds. In: Proceedings of the IEEE/CVF Conference on Computer Vision and Pattern Recognition, pp. 12697–12705 (2019)

[4] Shi, S., Wang, X., Li, H.: Pointrcnn: 3d object proposal generation and detection from point cloud. In: Proceedings of the IEEE/CVF Conference on Computer Vision and Pattern Recognition, pp. 770–779 (2019)

[5] Shi, S., Wang, Z., Shi, J., Wang, X., Li, H.: From points to parts: 3d object detection from point cloud with part-aware and part-aggregation network. IEEE transactions on pattern analysis and machine intelligence **43**(8), 2647–2664 (2020)

[6] Ren, Y., Zhu, F., Liu, W., Wang, Z., Lin, Y., Gao, F., Zhang, F.: Bubble planner: Planning high-speed smooth quadrotor trajectories using receding corridors. In: 2022 IEEE/RSJ International Conference on Intelligent Robots and Systems (IROS), pp. 6332–6339 (2022). IEEE

[7] Tordesillas, J., Lopez, B.T., Everett, M., How, J.P.: Faster: Fast and safe trajectory planner for navigation in unknown environments. IEEE Transactions on Robotics **38**(2), 922–938 (2021)

[8] Liu, Z., Zhang, F., Hong, X.: Low-cost retina-like robotic lidars based on incommensurable scanning. IEEE/ASME Transactions on Mechatronics **27**(1), 58–68 (2021)

[9] Wang, D., Watkins, C., Xie, H.: Mems mirrors for lidar: A review. Micromachines **11**(5), 456 (2020)

[10] Dewan, A., Caselitz, T., Tipaldi, G.D., Burgard, W.: Rigid scene flow for 3d lidar scans. In: 2016 IEEE/RSJ International Conference on Intelligent Robots and Systems (IROS), pp. 1765–1770 (2016). IEEE

[11] Pontes, J.K., Hays, J., Lucey, S.: Scene flow from point clouds with or without learning. In: 2020 International Conference on 3D Vision (3DV), pp. 261–270 (2020). IEEE

[12] Li, X., Kaesemodel Pontes, J., Lucey, S.: Neural scene flow prior. Advances in Neural Information Processing Systems **34**, 7838–7851 (2021)

[13] Liu, X., Qi, C.R., Guibas, L.J.: Flownet3d: Learning scene flow in 3d point clouds. In: Proceedings of the IEEE/CVF Conference on Computer Vision and Pattern Recognition, pp. 529–537 (2019)

[14] Behl, A., Paschalidou, D., Donné, S., Geiger, A.: Pointflownet: Learning representations for rigid motion estimation from point clouds. In: Proceedings of the IEEE/CVF Conference on Computer Vision and Pattern Recognition, pp. 7962–7971 (2019)

[15] Wang, Z., Li, S., Howard-Jenkins, H., Prisacariu, V., Chen, M.: Flownet3d++: Geometric losses for deep scene flow estimation. In: Proceedings of the IEEE/CVF Winter Conference on Applications of Computer Vision, pp. 91–98 (2020)

[16] Ushani, A.K., Wolcott, R.W., Walls, J.M., Eustice, R.M.: A learning approach for real-time temporal scene flow estimation from lidar data. In: 2017 IEEE International Conference on Robotics and Automation (ICRA), pp. 5666–5673 (2017). IEEE

[17] Wu, W., Wang, Z.Y., Li, Z., Liu, W., Fuxin, L.: Pointpwc-net: Cost volume on point clouds for (self-) supervised scene flow estimation. In: European Conference on Computer Vision, pp. 88–107 (2020). Springer

[18] Mittal, H., Okorn, B., Held, D.: Just go with the flow: Self-supervised scene flow estimation. In: Proceedings of the IEEE/CVF Conference on Computer Vision and Pattern Recognition, pp. 11177–11185 (2020)

[19] Milioto, A., Vizzo, I., Behley, J., Stachniss, C.: Rangenet++: Fast and accurate lidar semantic segmentation. In: 2019 IEEE/RSJ International Conference on Intelligent Robots and Systems (IROS), pp. 4213–4220 (2019). IEEE

[20] Cortinhal, T., Tzelepis, G., Erdal Aksoy, E.: Salsanext: Fast, uncertainty-aware semantic segmentation of lidar point clouds. In: International Symposium on Visual Computing, pp. 207–222 (2020). Springer

[21] Chen, X., Li, S., Mersch, B., Wiesmann, L., Gall, J., Behley, J., Stachniss, C.: Moving object segmentation in 3d lidar data: A learning-based approach exploiting sequential data. IEEE Robotics and Automation Letters **6**(4), 6529–6536 (2021)

[22] MAVROS. https://github.com/mavlink/mavros

[23] He, D., Xu, W., Zhang, F.: Symbolic representation and toolkit development of iterated error-state extended kalman filters on manifolds. IEEE Transactions on Industrial Electronics (2023)

[24] Xu, W., Cai, Y., He, D., Lin, J., Zhang, F.: Fast-lio2: Fast direct lidar-inertial odometry. IEEE Transactions on Robotics (2022)

[25] Ester, M., Kriegel, H.-P., Sander, J., Xu, X., *et al.*: A density-based algorithm for discovering clusters in large spatial databases with noise. In: Kdd, vol. 96, pp. 226–231 (1996)

[26] Kong, F., Xu, W., Cai, Y., Zhang, F.: Avoiding dynamic small obstacles with onboard sensing and computation on aerial robots. IEEE Robotics and Automation Letters **6**(4), 7869–7876 (2021)

[27] Mellinger, D., Kumar, V.: Minimum snap trajectory generation and control for quadrotors. In: 2011 IEEE International Conference on Robotics and Automation, pp. 2520–2525 (2011). IEEE

[28] Kuhn, H.W.: The hungarian method for the assignment problem. Naval research logistics quarterly **2**(1-2), 83–97 (1955)
