# Supplementary material for: Moving event detection from LiDAR point streams
Source: Nat Commun. 2024 Jan 6;15:345. doi: 10.1038/s41467-023-44554-8 (PMC10771495; doi:10.1038/s41467-023-44554-8)
Supplement: Supplementary file 3 — Description of Additional Supplementary Files [file 41467_2023_44554_MOESM3_ESM.docx]

**Description of Additional Supplementary Files**

**Supplementary Video 1:** Robust detection of M-detector in a complicated scene.

**Supplementary Video 2:** The application of M-detector on UAV obstacle avoidance.

**Supplementary Video 3:** The application of M-detector on traffic monitoring.

**Supplementary Video 4:** The application of M-detector on surveillance.

**Supplementary Video 5:** The application of M-detector on mapping.

**Supplementary Video 6:** Overview of M-detector.
